# Improving Clinical Outcomes in Newly Diagnosed Pediatric Type 1 Diabetes: Teamwork, Targets, Technology, and Tight Control—The 4T Study

**DOI:** 10.3389/fendo.2020.00360

**Published:** 2020-07-09

**Authors:** Priya Prahalad, Dessi P. Zaharieva, Ananta Addala, Christin New, David Scheinker, Manisha Desai, Korey K. Hood, David M. Maahs

**Affiliations:** ^1^Division of Endocrinology, Department of Pediatrics, Stanford University, Stanford, CA, United States; ^2^Department of Management Science and Engineering, Stanford University, Stanford, CA, United States; ^3^Quantitative Sciences Unit, Division of Biomedical Informatics Research, Stanford University, Stanford, CA, United States; ^4^Stanford Diabetes Research Center, Stanford, CA, United States

**Keywords:** type 1 diabetes, pediatrics, insulin pump, continuous glucose monitoring, hemoglobin A1c

## Abstract

Many youth with type 1 diabetes (T1D) do not achieve hemoglobin A1c (HbA1c) targets. The mean HbA1c of youth in the USA is higher than much of the developed world. Mean HbA1c in other nations has been successfully modified following benchmarking and quality improvement methods. In this review, we describe the novel 4T approach—teamwork, targets, technology, and tight control—to diabetes management in youth with new-onset T1D. In this program, the diabetes care team (physicians, nurse practitioners, certified diabetes educators, dieticians, social workers, psychologists, and exercise physiologists) work closely to deliver diabetes education from diagnosis. Part of the education curriculum involves early integration of technology, specifically continuous glucose monitoring (CGM), and developing a curriculum around using the CGM to maintain tight control and optimize quality of life.

## Introduction

The Diabetes Control and Complications Trial (DCCT) firmly established the efficacy of intensive diabetes management to reduce vascular complications of type 1 diabetes (T1D) (1, 2). Despite results from the DCCT, most youth do not meet glycemic targets (3). For example, based on the T1D Exchange (T1DX) registry, the mean hemoglobin A1c (HbA1c) in 17-year-old Americans in 2010–2012 is similar to the conventional arm of the DCCT (9%) (1, 2, 4), whereas in the DPV registry in Germany and Austria, the mean HbA1c is 8.2% (5). Current care for children, adolescents, and young adults with T1D has failed to make meaningful progress in lowering HbA1c despite advances in diabetes technology (6–8) [insulin pumps, continuous glucose monitoring (CGM), and now automated insulin delivery systems, analog insulins (both basal and bolus), and refinements in care delivery, among others] (9).

There have been efforts to modify care delivery to improve outcomes in individuals with T1D. For example, in the DPV registry in Germany and Austria, HbA1c decreased from 9 to 8.2% from 1995 to 2010 on the basis of benchmarking and quality improvement methods (10, 11). The comparison of 2015 HbA1c data between the T1DX and DPV shows about a 1% gap in people 3–21 years of age (12). Similarly, numerous other countries with similar economic status to the USA have reported reductions in HbA1c, leaving the mean HbA1c in American youth with T1D among the highest worldwide (3). While these countries have dramatically different health-care systems, all are in developed countries such as the USA. In contrast, the international SWEET registry reports the mean HbA1c in individual clinics in many developing countries such as India, Nepal, and Mexico are in the 8–9.5% range, suggesting that even in resource-poor situations, better glucose control than the USA can be achieved (13). Therefore, other countries have effectively implemented the DCCT message of intensive glucose control, leaving the USA as an outlier in achieved pediatric HbA1c (14–16). In fact, pediatric HbA1c in the T1DX was higher in the 2017 data than in 2010–2012 data (17). From literature describing efforts to decrease HbA1c (10, 18), common themes emerge as potentially critical contributors to success in HbA1c management (9, 19, 20). These include (1) a unified and consistent team approach; (2) communicating clear glucose targets to youth and their families; (3) flexibility in supporting youth and families; and (4) timely detection of increasing glucose trends followed by rapid intensification of therapy to regain target control.

Moreover, a practical and sustainable approach to improving long-term outcomes is to focus on youth with new-onset T1D. A recent study has shown that an individual's long-term glycemic track is set by 5 years post-diabetes diagnosis (21), and we have previously shown that HbA1c rises between 5 and 6 months post-diabetes diagnosis and levels at 12–18 months (22). Taken together, these studies suggest that interventions early in the course of diabetes can have long-term impact on glycemic outcomes. Additional rationale to focus on newly diagnosed youth includes the following: a fresh start to deliver diabetes education and establishing diabetes care habits instead of reteaching and breaking old habits, both for youth and providers; greater efficiency in the use of resources to maintain rather than regain tight control; and the opportunity to capitalize on the tighter control that commonly occurs post-T1D diagnosis. In this review, we will describe the 4T (teamwork, targets, technology, and tight control) approach to improving outcomes in youth with T1D.

## The 4T Approach

Increased frequency of blood glucose monitoring has been shown to improve HbA1c (23). Glucometers provide an intermittent trend of glucoses, but CGM, which measures 96–288 glucose values per day, also provides glucose trends. Given the benefit of CGM, the cornerstone of the 4T approach is to start youth on CGM technology within the first month of diagnosis to allow for tighter control of glucoses in the new-onset period. Initiation of CGM so early in the course of T1D requires highly coordinated teamwork to provide the education needed for youth and families to manage not only diabetes but also the large volume of data provided by the CGM. Using technology and creating an education curriculum centered on the data provided by CGM can allow for tighter targets during the honeymoon period and beyond. The main principle behind the 4T approach is that using a CGM is not sufficient. The data must be frequently reviewed and dose adjustments or additional education should be provided to maintain glucoses in the target range. To prevent overburdening the care team with data analysis, clinical decision support tools are necessary to automate data analysis and facilitate population health management.

### 4T Protocol

Historically, our new-onset care involved a 4- to 6-h diabetes education visit with the care team [physician, certified diabetes educator (CDE), and registered dietician]. Patients received daily phone calls from the care team for dose adjustments until the recent onset visit, which occurred at 1 week post-diagnosis. There was another follow-up visit at 1 month post-diagnosis followed by routine follow-up every 3 months. Patients could start on diabetes technology at various time points in this process.

**Figure 1:**
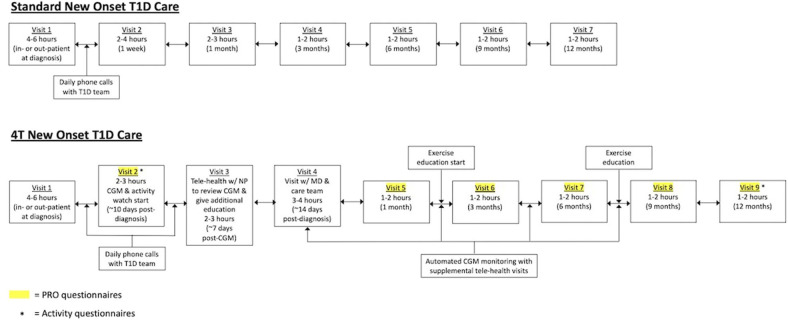
Standard new-onset type 1 diabetes (T1D) care compared with the 4T new-onset T1D program.

In the 4T program (Figure 1), we are revising our new-onset program to provide more touchpoints between patients with newly diagnosed T1D and the care team. In addition, technology, in the form of a Dexcom G6 CGM, is introduced in the first month post-diagnosis. Following routine new-onset education, all patients with newly diagnosed T1D are offered the opportunity to initiate CGM within the first month of diagnosis. Individuals who choose to participate in this study will receive CGM through the study for 1 year. CGM initiation will occur at an additional visit that is typically 10 days post-diagnosis. Patients will continue to receive daily phone calls from the diabetes care team until a telehealth education visit with a nurse practitioner, which typically occurs 1 week after the CGM start. Patients then have subsequent follow-ups at 2 weeks post-diagnosis, 1 month post-diagnosis, and then every 3 months. In addition, individuals can elect to be part of a remote monitoring arm in which CGM data are reviewed by the care team weekly following CGM initiation. The care team will reach out to families through secure messaging, phone calls, and telehealth visits to provide education and dose adjustments between visits. All patients are started on a multidose injection (MDI) regimen at diagnosis. However, there are no restrictions on adding other pieces of diabetes technology including insulin pumps or hybrid closed-loop systems. At approximately, 1 month post-diagnosis, patients can also elect to be part of an exercise intervention. Patient-reported outcomes (PROs) will be obtained at visits 2 and 5–9.

### Technology

Recent advances in diabetes technology place us in the third era of glucose monitoring (urine, blood, and now interstitial fluid with CGM systems). Compared with self-monitoring of blood glucose (SMBG), CGM has the advantages of providing readings as frequently as every 5 min (up to 288 readings per day) with fewer finger pricks as well as providing the rate and direction of glucose change. Rates of CGM use in pediatrics as reported by the T1DX have increased from 4% (2013) to 14% (2015) to 31% (2017) (24–26), similar to what is observed worldwide (24, 26). Retrospective studies examining the use of CGM in the first year of diabetes diagnosis have demonstrated improvements in glycemic control (27, 28). However, in these studies, the timing of CGM initiation has been variable, and many of the youth started after the rise in HbA1c observed between 5 and 6 months post-diagnosis. Initiation of CGM in the first month of diabetes diagnosis can allow for more frequent insulin dose adjustments in between visits and education around tighter targets. In a pilot study, our team initiated 40 youth on CGM in the first month of diagnosis and demonstrated a high persistence of use with a low incidence of hypoglycemia in participants (29).

**Figure 2:**
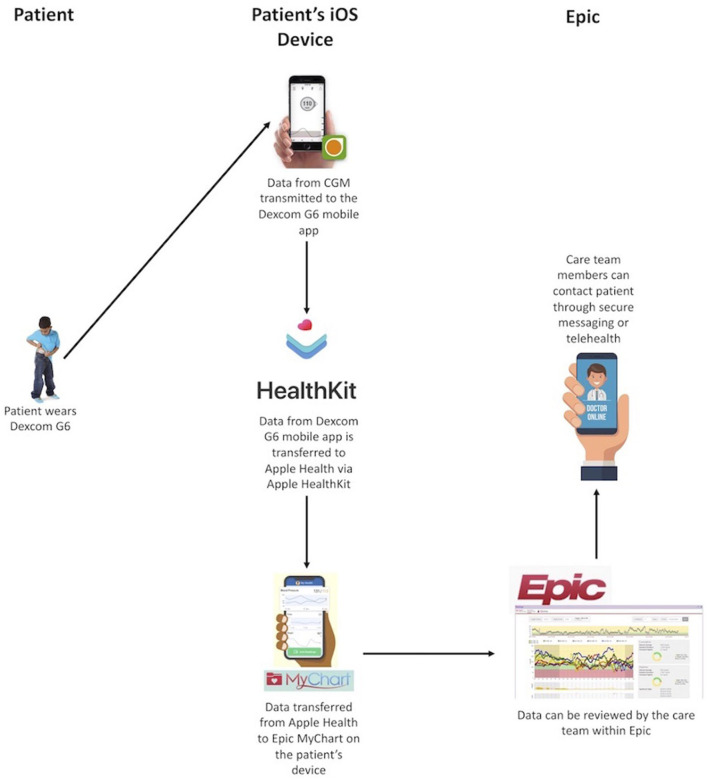
Integration of Dexcom CGM data into the electronic health record.

In the past year, CGM systems have been approved for use in insulin dosing (non-adjunctive use) and for factory calibration, allowing it to be a true substitute for SMBG. These features combined with the increased number of readings, rates and directions of change, alerts, and remote data sharing have promise for improving glycemic control in youth with T1D. The remote data sharing feature of CGM can allow for remote monitoring not just by a youth's caregiver but also the youth's care team. We have previously developed a system for transmitting data from an individual's CGM into our electronic health record (EHR) using Apple HealthKit on iOS devices [Figure 2; (30)]. This system meets criteria set forth by the US Health Information Portability and Accountability Act (HIPAA). With the use of this feature, CGM data can be reviewed by the diabetes care team, securely, to perform dose adjustments in between clinic visits. Dose adjustments are performed using a secure patient messaging platform embedded in the EHR or via secure telehealth, also embedded in the EHR. Given the large number of individuals in any clinic population, population health management tools need to be developed to allow for the frequent review of a large volume of data. Unfortunately, the increased use of CGM in pediatric T1D care has not been fully utilized in how education is provided, targets are defined, or glucose data are used in between quarterly clinic visits.

Other technologies can also change diabetes care delivery. Internet access, including smart phone usage, is becoming nearly ubiquitous, even in the developing world (31). The high penetrance of internet access can allow for remote care delivery through telehealth.

Standard of care for individuals with T1D is at least four visits per year with a HbA1c measurement at each visit. Children who attend quarterly visits were more likely to have improved HbA1c than those attending fewer visits per year (32). Data suggest that children who were further from their diabetes center had fewer visits per year and a higher HbA1c (33). In our practice, we have youth who drive several hundred miles for care, which makes clinical visits burdensome and expensive. Telehealth provided to an individual's home allows for face-to-face care without parents missing work, children missing school, or traveling. Telehealth can also be utilized for group education classes to reinforce diabetes learnings. Because telehealth visits do not require dedicated clinic space or ancillary staff, visit times can be more flexible to meet the needs of youth and their caregivers.

Technology has the potential to improve care delivery to people with T1D. However, the limiting resource is the time of the care team. Modern data analysis and visualization tools allow for the design of decision-support tools that translate the analysis of CGM data to allow for efficient population-health management with existing staff.

### Teamwork

Care for youth with T1D requires a coordinated effort by a team consisting of pediatric endocrinologists, CDEs, nurses, registered dieticians, social workers, psychologists, and exercise physiologists. Both the American Diabetes Association (ADA) (34) and the International Society for Pediatric and Adolescent Diabetes (ISPAD) (35) advocate for a team-based approach to diabetes management. The team-based approach starts in the new-onset period, with education provided to the family by physicians, diabetes educators, and registered dieticians. Initiation of CGM requires a highly integrated team effort to not only provide basic diabetes education but also to provide education on using CGM for improved diabetes care. The role of the diabetes care team does not end in the new-onset period and should be ongoing. Thus, access to these care team members should be available at each follow-up visit.

In addition to education, T1D is associated with psychosocial comorbidities. In a systematic review, one third of youth with T1D experienced diabetes distress, which is the emotional distress that is associated with the burden of diabetes self-management (36). Diabetes distress is associated with poorer clinical outcomes. Given the importance of mental health to the care of children and young adults with T1D, mental health support, through psychologists and social workers, should be made available to help with diabetes management. PROs have been an established means of screening for psychosocial issues as recommended by the ADA guidelines.

In the 4T program, all youth in the clinic will receive early psychoeducation that describes symptoms of common psychological concerns in youth with T1D (e.g., distress and depression). This will be administered as part of standard teaching visits. All youth will be screened regularly (visits 2, 4, 5, 6, and 7), and therapeutic services can be offered when there are positive screens on the PROs (Table 1). Several examples of the interventions delivered as part of the 4T program include the Guiding Adolescents Through E-Psychotherapy (GATE) program, a five-session web-delivered cognitive behavioral therapy (CBT) program that is tailored to diabetes-specific concerns and designed to address topics that are most salient in these youths' lives. CBT is a gold-standard psychotherapy treatment for depression and the type of psychotherapy treatment that pediatric youth are most likely to receive in the community (41, 42). Not all youth will be able to attend weekly psychotherapy sessions; therefore, psychotherapy will be delivered via telehealth. Previous literature has shown this to be effective (43–45).

**Table 1:** PROs incorporated into the 4T program.

**Topic**	**Construct measured/relevant points**
**Youth and parent outcomes—diabetes distress**	
Diabetes distress (Diabetes Distress Scale) Fisher et al. (37)	The DDS-2 (2 item youth survey) and DDS-P (20 item parent survey) are commonly used measures to understand distress symptoms related to diabetes. Validated and used in >25 publications. The DDS-2 will be given to those above 11 years old. DDS-P will be given to all parents. The surveys take 5 min to complete.
**Youth-only outcomes**	
Diabetes technology attitudes Naranjo et al. (38)	Subjective questions about attitudes related to diabetes technologies and devices. Predictive of outcomes and interacts with diabetes distress. Has 6 items; 2 min to complete.
PROMIS Global Health (7-item Global Health) Forrest et al. (39)	This survey was developed by the PROMIS team and measures aspects of physical and mental health; 7 items and 3 min to complete. We have found it to be sensitive to picking up general quality of life changes.
CGM Benefits and Burden Scale Messer et al. (40)	This survey is a 16-item survey that evaluates perceived benefits (8 items) and burden (8 items) specific to CGM use.

*PROs, patient-reported outcomes*.

### Targets

Advice and teaching on glucose targets should be updated in response to existing diabetes technology, specifically the availability of CGM systems that provide real-time glucose data every 5 minutes, trend arrows, and alarms to notify youth and parents (remotely, if desired) when thresholds are crossed. A previous study has demonstrated the acceptability of CGM to both parents and youth soon after diagnosis via a “CGM at Diagnosis” protocol with 55 newly diagnosed pediatric youth (46). An important finding of the CGM group (vs. no-CGM control group) was the benefit of three times less hypoglycemia, and parents reported more confidence in managing hypoglycemia when they used CGM in the first 6 months after diagnosis (47). Therefore, CGM provides a safety net to allow for tighter glucose targets especially after new onset and has psychosocial benefits. Glucose targets, however, have not been adapted to aim for tighter control, a missed opportunity to lower HbA1c, although data are clear on the effectiveness of clear target setting (48). Three sets of HbA1c and related glucose targets have been published [Table 2; (51–53)]. The 4T approach involves educating families on the association of glucose data with specific HbA1c targets (50). Initial targets after diagnosis will be an HbA1c of 6.5%, and this target should be adapted and individualized to each family per guideline recommendations but in a standardized manner with a team approach. Recent data indicate that glucose time-in-range (TIR) can also be a useful metric for families that correlate with HbA1c (49). Unlike HbA1c, which is measured quarterly, TIR (and mean CGM glucose) can be determined between clinic visits and provide guidance on glucose control and insulin dosing (54, 55). Moreover, we will emphasize education to differentiate between hypoglycemia as a clinical alert of hypoglycemia (stage 1, 70–54 mg/dl) and clinically important or serious hypoglycemia (stage 2, <54 mg/dl) or seizure/coma (stage 3) (55). Each youth and family will receive HbA1c, mean glucose, TIR, and hypoglycemia targets to guide care. Simple metrics for education include aiming for a mean CGM glucose of 150 mg/dl, 70% of time spent between 70 and 180 mg/dl, <4% of time below 70 mg/dl, and <1% of time below 54 mg/dl or the shorthand phrasing: 150/70/4/1. Families will be provided education on interpreting CGM data and performing basic insulin dose adjustments.

**Table 2:** Glucose targets from NICE, ISPAD, and ADA (49–51).

	**NICE Goal A1c (49)** **≤48 mmol/mol (≤6.5%)**	**ISPAD Goal A1c (50)** **<53 mmol/mol (<7.0%)**	**ADA Goal A1c (51)** **<58 mmol/mol (<7.5%)**
Pre-meal	4.0–7.0 mmol/l (70–126 mg/dl)	4.0–7.2 mmol/l (70–130 mg/dl)	5.0–7.2 mmol/l (90–130 mg/dl)
Post-meal	5.0–9.0 mmol/l (90–162 mg/dl)	5.0–10.0 mmol/l (90–180 mg/dl)	
Pre-bed	4.0–7.0 mmol/l (70–126 mg/dl)	4.4–7.8 mmol/l (80–140 mg/dl)	5.0–8.3 mmol/l (90–150 mg/dl)

*NICE, National Institute for Health and Care Excellence; ISPAD, International Society for Pediatric and Adolescent Diabetes; ADA, American Diabetes Association*.

### Tight Control

Frequent review of data and dose adjustments are central to maintaining tight control. Unfortunately, only a minority of youth or caregivers review diabetes device data on a regular basis (56). Typically, the diabetes care team reviews glucoses every 3 months at patient visits. This interval may be too long to optimize glycemic control, especially in a growing child and one who is recently diagnosed. Several CGM systems offer the ability to share data remotely through mobile apps. This ability can be used by diabetes care teams to perform remote data review and dose adjustments for youth with T1D in between clinic visits rather than relying on HbA1c values, which reflect 3 months of glucose control (57, 58). This creates the opportunity to intervene in response to problematic trends as soon as they arise, rather than only when clinical care occurs at 3-month intervals. Given the volume of data, performing routine reviews for all youth can be burdensome to the diabetes care team. As a result, tools should be developed to facilitate population health management.

Recent applications of machine learning and decision support have shown great promise for informing clinical decisions, but not (59) to developing personalized disease management recommendations for youth with T1D (59–61). We have developed a system that aims to identify deviations and opportunities at shorter intervals to notify the clinical team for the possible need for insulin adjustments. The system is designed with equal emphasis on algorithmic analysis of CGM data and on facilitating a systematic, coordinated approach by the care team. The algorithms identify youth with deteriorating control and generate alerts. The systematic, coordinated approach ensures that care team members can (1) consistently see data for a small cohort of “their” youth; (2) quickly review data for all other youth for whom there has been an alert; and (3) maximize efficiency by only reviewing data that have not already been reviewed by someone else or contacting a patient that has been recently contacted. This will allow care team members to prioritize data review of individuals who require additional interventions to maintain tight control, thereby decreasing the burden on care team members.

### Exercise in Youth With New-Onset Type 1 Diabetes

Regular physical activity is important to promote well-being, psychological development, and overall health (62–64). However, it is also recognized that many individuals with T1D are not engaging in the recommended daily levels of physical activity (65). Youth living with T1D and health-care professionals have identified “exercising safely” as one of the most challenging aspects of diabetes, and many choose not to partake in activity because of the associated risks. Matson et al. (66) reported that newly diagnosed adults with T1D spent on average a quarter less time in moderate-to-vigorous physical activity (MVPA) per day than did adults without T1D. Similarly, for children who develop T1D under the age of 7, the rates of physical activity are significantly lower than in those without T1D (67). These lower rates of activity may also be due to factors including patient fear of hypoglycemia and primary caregiver restrictions (68).

For youth with T1D, many factors need to be taken into consideration before engaging in physical activity such as starting glucose level, timing and intensity of activity, insulin dosing, carbohydrate supplementation, exercise time of day, individual fitness, and prior episodes of hypoglycemia (69–71). It is essential for clinicians and health-care practitioners to encourage regular physical activity; however, this requires a more in-depth understanding of strategies to better manage glycemia during exercise (66, 70). Clinical guidelines, consensus, and position statements on exercise provide some structured approaches to assist clinicians in forming individualized exercise management plans for youth with T1D (53, 70, 72). To reduce exercise-associated hypoglycemia, some strategies include reducing the basal insulin dose pre-exercise, reducing prandial insulin for the meal before exercise, and/or increasing carbohydrate feeding (53). Overall, more vigilant and frequent monitoring of blood glucose around exercise is recommended for safety.

In an attempt to achieve tighter glucose targets in the 4T study, an additional focus will include exercise education. The teaching materials will be generated using current international findings and published research. The aim is to provide this education material to youth and their families in the first 12 months of diabetes duration, as this is where the rise in HbA1c has been previously documented.

### Preliminary Outcomes

Since July 2018 and as of December 2019, we have had 90 youth initiate CGM in the new-onset period. Of those 90 youth, 65 of them have been in this program for at least 6 months. Although the median HbA1c at diabetes onset was higher relative to our previous cohort from 2014 to 2016, the nadir was lower. At 6 months post-diabetes diagnosis, unadjusted the HbA1c was 0.54% lower in the new-onset CGM cohort compared with our historic controls (73).

Of the 65 participants, 30 were enrolled in a remote monitoring study, facilitated through an internal grant, whereby participants were provided an iOS device if they did not have one of their own. We integrated their data into our EHR (Figure 2). Data were reviewed weekly by a member of the diabetes care team and youth and/or caregivers were contacted for insulin dose adjustments and/or education. On average, these youth had 15 data reviews between clinic visits with an average of five dose changes per patient (74). Efforts to streamline this process are ongoing so that it can be scaled to a larger population without increasing burden on the diabetes care team.

## Conclusions

Despite the results of the DCCT, many youth with T1D do not meet glycemic targets. With benchmarking and quality improvement efforts, clinics have managed to lower HbA1c closer to the ISPAD target of 7%, but few have achieved this target. Although technology has helped to ease some of the burden related to T1D, it has not led to significant improvements in glycemic control. Some of the challenges lie in the fact that youth with T1D received education in the pre-CGM days when targets were not as tight to prevent severe hypoglycemia. CGM technology allows for tighter targets because there are alerts to prevent clinically significant hypoglycemia. In addition, automated insulin delivery systems in which CGM is an integral component use the CGM to suspend insulin for suspected hypoglycemia. Despite advances in technology, education has not evolved to promote tighter targets.

The 4T approach discussed in this review develops diabetes education and a management program centered around the use of technology, specifically CGM, in the new-onset period. The new-onset period was chosen because it offers the opportunity to provide education to youth with T1D and their caregivers, which is consistent with modern diabetes care. Initiation of CGM in the new-onset period requires a coordinated team effort by physicians, diabetes educators, nutritionists, social workers, exercise physiologists, and psychologists. Once CGM is initiated, education can focus around consistent targets to achieve tight control. Technology can also allow for remote monitoring of CGM data, and the development of population health tools can make CGM review between clinic visits the standard of care for all youth. With the advent of telehealth, care can be delivered by virtual teleconference, further decreasing the burden of diabetes care by decreasing travel to diabetes clinic.

Psychosocial support is an integral part of this program. Although CGM has the opportunity to decrease the burden of diabetes care and improve glycemic control, it should not come at a cost to quality of life. Thus, an important piece of the 4T program is to monitor PROs while also making psychosocial support a key element of diabetes care.

In conclusion, the 4T program aims to achieve tighter glucose control after T1D diagnosis and maintain it as the clinical remission phase wanes while optimizing quality of life. Moreover, in the future, automated insulin delivery systems promise tighter glucose control with less hypoglycemia and lower burden of care for youth with T1D and their families (75–78). Therefore, early integration and attention to the adoption of diabetes technology and psychosocial outcomes will be increasingly important to fully realize the potential of optimal T1D care.

## Author Contributions

PP, AA, DZ, DS, MD, KH, and DM contributed to the concept and design of the study. MD performed the statistical analysis. PP wrote the first draft of the manuscript. All authors contributed to manuscript revision and approved the submitted manuscript.

## Conflict of Interest

KH has research support from Dexcom, Inc., for investigator- initiated research and consultant fees from Lilly Innovation Center, Lifescan Diabetes Institute and MedIQ. DM has research support from the NIH, JDRF, NSF, and the Helmsley Charitable Trust; and his institution has research support from Medtronic, Dexcom, Insulet, Bigfoot Biomedical, Tandem, and Roche. DM has consulted for Abbott, the Helmsley Charitable Trust, Sanofi, Novo Nordisk, Eli Lilly, and Insulet. He is supported by P30DK116074. DZ has received speaker's honoraria from Medtronic Diabetes, Ascensia Diabetes, and Insulet Canada. MD has received support from Sanofi, Bayer, and the NIH. The remaining authors declare that the research was conducted in the absence of any commercial or financial relationships that could be construed as a potential conflict of interest.

## References

[1] Diabetes Control and Complications Trial Research Group Effect of intensive diabetes treatment on the development and progression of long-term complications in adolescents with insulin-dependent diabetes mellitus: diabetes control and complications trial. Diabetes Control and Complications Trial Research Group. J Pediatr. (1994) 125:177–88. 10.1016/S0022-3476(94)70190-3 8040759

[2] The Diabetes Control Complications Trial Research Group. The effect of intensive treatment of diabetes on the development and progression of long-term complications in insulin-dependent diabetes mellitus. N Engl J Med. (1993) 329:977–86. 10.1056/NEJM199309303291401 8366922

[3] Charalampopoulos D Hermann JM Svensson J Skrivarhaug T Maahs DM Akesson K. Exploring variation in glycemic control across and within eight high-income countries: a cross-sectional analysis of 64,666 children and adolescents with type 1 diabetes. Diabetes Care. (2018) 41:1180–7. 10.2337/dc17-2271 29650804 PMC5961394

[4] Miller KM Foster NC Beck RW Bergenstal RM Dubose SN Dimeglio LA. Current state of type 1 diabetes treatment in the U.S.: updated data from the T1D exchange clinic registry. Diabetes Care. (2015) 38:971–8. 10.2337/dc15-0078 25998289

[5] Hofer SE Raile K Frohlich-Reiterer E Kapellen T Dost A Rosenbauer J. Tracking of metabolic control from childhood to young adulthood in type 1 diabetes. J Pediatr. (2014) 165:956–61.e1–2. 10.1016/j.jpeds.2014.07.001 25151197

[6] Sherr JL Hermann JM Campbell F Foster NC Hofer SE Allgrove J. Use of insulin pump therapy in children and adolescents with type 1 diabetes and its impact on metabolic control: comparison of results from three large, transatlantic paediatric registries. Diabetologia. (2016) 59:87–91. 10.1007/s00125-015-3790-6 26546085

[7] Szypowska A Schwandt A Svensson J Shalitin S Cardona-Hernandez R Forsander G. Insulin pump therapy in children with type 1 diabetes: analysis of data from the SWEET registry. Pediatr Diabetes. (2016) 17(Suppl. 23):38–45. 10.1111/pedi.12416 27417128

[8] Prahalad P Tanenbaum M Hood K Maahs DM. Diabetes technology: improving care, improving patient-reported outcomes and preventing complications in young people with Type 1 diabetes. Diabet Med. (2018) 35:419–29. 10.1111/dme.13588 29356074

[9] Cameron FJ De Beaufort C Aanstoot HJ Hoey H Lange K Castano L. Lessons from the Hvidoere International Study Group on childhood diabetes: be dogmatic about outcome and flexible in approach. Pediatr Diabetes. (2013) 14:473–80. 10.1111/pedi.12036 23627895

[10] Rosenbauer J Dost A Karges B Hungele A Stahl A Bachle C. Improved metabolic control in children and adolescents with type 1 diabetes: a trend analysis using prospective multicenter data from Germany and Austria. Diabetes Care. (2012) 35:80–6. 10.2337/dc11-0993 22074726 PMC3241332

[11] Karges B Rosenbauer J Kapellen T Wagner VM Schober E Karges W. Hemoglobin A1c Levels and risk of severe hypoglycemia in children and young adults with type 1 diabetes from Germany and Austria: a trend analysis in a cohort of 37,539 patients between 1995 and 2012. PLoS Med. (2014) 11:e1001742. 10.1371/journal.pmed.1001742 25289645 PMC4188517

[12] Hermann JM Miller KM Hofer SE Clements MA Karges W Foster NC International Comparison of HbA1c Across Lifespan in Males and Females with Type 1 Diabetes. Chicago, IL: ADA (2020).

[13] Danne T Hanas R. The mission of SWEET: harmonize care to optimize outcomes of children with diabetes worldwide. Pediatr Diabetes. (2016) 17(Suppl. 23):3–6. 10.1111/pedi.12411 27748025

[14] Dovc K Telic SS Lusa L Bratanic N Zerjav-Tansek M Kotnik P. Improved metabolic control in pediatric patients with type 1 diabetes: a nationwide prospective 12-year time trends analysis. Diabetes Technol Ther. (2014) 16:33–40. 10.1089/dia.2013.0182 24131373 PMC3887404

[15] Samuelsson U Akesson K Peterson A Hanas R Hanberger L. Continued improvement of metabolic control in Swedish pediatric diabetes care. Pediatr Diabetes. (2018) 19:150–7. 10.1111/pedi.12467 27807917

[16] Sumnik Z Venhacova J Skvor J Pomahacova R Konecna P Neumann D. Five years of improving diabetes control in Czech children after the establishment of the population-based childhood diabetes register CENDA. Pediatr Diabetes. (2020) 21:77–87. 10.1111/pedi.12929 31605416

[17] Foster NC Beck RW Miller KM Clements MA Rickels MR Dimeglio LA. State of type 1 diabetes management and outcomes from the T1D exchange in 2016-2018. Diabetes Technol Ther. (2019) 21:66–72. 10.1089/dia.2018.0384 30657336 PMC7061293

[18] Karges B Schwandt A Heidtmann B Kordonouri O Binder E Schierloh U Association of insulin pump therapy vs insulin injection therapy with severe hypoglycemia, ketoacidosis, and glycemic control among children, adolescents, and young adults with type 1 diabetes. JAMA. (2017) 318:1358–66. 10.1001/jama.2017.13994 29049584 PMC5818842

[19] Wood JR Miller KM Maahs DM Beck RW Dimeglio LA Libman IM Most youth with type 1 diabetes in the T1D exchange clinic registry do not meet American Diabetes Association or International Society for pediatric and adolescent diabetes clinical guidelines. Diabetes Care. (2013) 36:2035–7. 10.2337/dc12-1959 23340893 PMC3687259

[20] Skinner TC Lange KS Hoey H Mortensen HB Aanstoot HJ Castano L. Targets and teamwork: understanding differences in pediatric diabetes centers treatment outcomes. Pediatr Diabetes. (2018) 19:559–65. 10.1111/pedi.12606 29159931

[21] Nirantharakumar K Mohammed N Toulis KA Thomas GN Narendran P Clinically meaningful and lasting HbA1c improvement rarely occurs after 5 years of type 1 diabetes: an argument for early, targeted and aggressive intervention following diagnosis. Diabetologia. (2018) 61:1064–70. 10.1007/s00125-018-4574-6 29478098 PMC6448997

[22] Prahalad P Yang J Scheinker D Desai M Hood K Maahs D. Hemoglobin A1c trajectory in pediatric patients with newly diagnosed type 1 diabetes. Diabetes Technol Therapeutics. (2019) 21:456–61. 10.1089/dia.2019.0065 31180244 PMC7001422

[23] Miller KM Beck RW Bergenstal RM Goland RS Haller MJ Mcgill JB Evidence of a strong association between frequency of self-monitoring of blood glucose and hemoglobin A1c levels in T1D exchange clinic registry participants. Diabetes Care. (2013) 36:2009–14. 10.2337/dc12-1770 23378621 PMC3687326

[24] DeSalvo D Miller KM Hermann JM Maahs DM Hofer SE Clements MA. Continuous Glucose Monitoring (CGM) and Glycemic Control Among Youth with Type 1 Diabetes (T1D): International comparison from the T1D Exchange and DPV Initiative. Pediatr Diabetes. (2018) 19:1271–5. 10.1111/pedi.12711 29923262 PMC6175652

[25] Miller KM Hermann JM Maahs DM Hofer SE Foster N Holl RW Holl for the T1D exchange and DPV registries. In: Increasing Use of Continuous Glucose Monitoring (CGM) Among Youth with Type 1 Diabetes (T1D): International Comparison of Youth from the T1D Exchange (T1DX) and the DPV Initiative (2018). 10.1111/pedi.12711 PMC6175652 29923262

[26] Miller KM Hermann J Foster N Hofer SE Rickels MR Danne T. Longitudinal changes in continuous glucose monitoring use among individuals with type 1 diabetes: international comparison in the german and Austrian DPV and U.S. T1D exchange registries. Diabetes Care. (2020) 43:e1–2. 10.2337/dc19-1214 31672703 PMC7881298

[27] Mulinacci G Alonso GT Snell-Bergeon JK Shah VN. Glycemic outcomes with early initiation of continuous glucose monitoring system in recently diagnosed patients with type 1 diabetes. Diabetes Technol Ther. (2019) 21:6–10. 10.1089/dia.2018.0257 30575413

[28] Patton SR Noser AE Youngkin EM Majidi S Clements MA. Early initiation of diabetes devices relates to improved glycemic control in children with recent-onset type 1 diabetes mellitus. Diabetes Technol Ther. (2019) 21:379–84. 10.1089/dia.2019.0026 31166808 PMC6602098

[29] Prahalad P Addala A Scheinker D Hood KK Maahs DM. CGM initiation soon after type 1 diabetes diagnosis results in sustained CGM use and wear time. Diabetes Care. (2019) 43:e3–e4. 10.2337/dc19-1205 31558548 PMC7011198

[30] Kumar RB Goren ND Stark DE Wall DP Longhurst CA. Automated integration of continuous glucose monitor data in the electronic health record using consumer technology. J Am Med Inform Assoc. (2016) 23:532–7. 10.1093/jamia/ocv206 27018263 PMC4901382

[31] Silver L. AS Smith A Johnson C Jiang J Anderson M Use of Smartphones and Social Media is Common Across Most Emerging Economies. (2019). Available: https://www.pewresearch.org/internet/2019/03/07/use-of-smartphones-and-social-media-is-common-across-most-emerging-economies/ (accessed December 9, 2019).

[32] Phan TL Hossain J Lawless S Werk LN. Quarterly visits with glycated hemoglobin monitoring: the sweet spot for glycemic control in youth with type 1 diabetes. Diabetes Care. (2014) 37:341–5. 10.2337/dc13-0980 24062334

[33] Fox DA Islam N Amed S. Type 1 diabetes outcomes: does distance to clinic matter? Pediatr Diabetes. (2018) 19:1331–6. 10.1111/pedi.12749 30101515

[34] American Diabetes Association 12. Children and adolescents. Diabetes Care. (2017) 40:S105–13. 10.2337/dc17-S015 27979899

[35] Pihoker C Forsander G Fantahun B Virmani A Corathers S Benitez-Aguirre P. ISPAD clinical practice consensus guidelines 2018: the delivery of ambulatory diabetes care to children and adolescents with diabetes. Pediatr Diabetes. (2018) 19(Suppl. 27):84–104. 10.1111/pedi.12757 30144259

[36] Hagger V Hendrieckx C Sturt J Skinner TC Speight J. Diabetes distress among adolescents with type 1 diabetes: a systematic review. Curr Diabetes Rep. (2016) 16:9. 10.1007/s11892-015-0694-2 26748793

[37] Fisher L Hessler DM Polonsky WH Mullan J. When is diabetes distress clinically meaningful?: establishing cut points for the Diabetes Distress Scale. Diabetes Care. (2012) 35:259–64. 10.2337/dc11-1572 22228744 PMC3263871

[38] Naranjo D Tanenbaum ML Iturralde E Hood KK. Diabetes technology: uptake, outcomes, barriers, and the intersection with distress. J Diabetes Sci Technol. (2016) 10:852–8. 10.1177/1932296816650900 27234809 PMC4928242

[39] Forrest CB Bevans KB Pratiwadi R Moon J Teneralli RE Minton JM. Development of the PROMIS (R) pediatric global health (PGH-7) measure. Qual Life Res. (2014) 23:1221–31. 10.1007/s11136-013-0581-8 24264804 PMC3966936

[40] Messer LH Cook PF Tanenbaum ML Hanes S Driscoll KA Hood KK. CGM benefits and burdens: two brief measures of continuous glucose monitoring. J Diabetes Sci Technol. (2019) 13:1135–41. 10.1177/1932296819832909 30854886 PMC6835174

[41] Juvenile Diabetes Research Foundation Continuous Glucose Monitoring Study Group. Effectiveness of continuous glucose monitoring in a clinical care environment: evidence from the Juvenile Diabetes Research Foundation continuous glucose monitoring (JDRF-CGM) trial. Diabetes Care. (2010) 33:17–22. 10.2337/dc09-1502 19837791 PMC2797966

[42] Edge J Acerini C Campbell F Hamilton-Shield J Moudiotis C Rahman S. An alternative sensor-based method for glucose monitoring in children and young people with diabetes. Arch Dis Child. (2017) 102:543–9. 10.1136/archdischild-2016-311530 28137708 PMC5466923

[43] van Bastelaar KM Pouwer F Cuijpers P Riper H Snoek FJ. Web-based depression treatment for type 1 and type 2 diabetic patients: a randomized, controlled trial. Diabetes Care. (2011) 34:320–5. 10.2337/dc10-1248 21216855 PMC3024341

[44] Ebert DD Zarski AC Christensen H Stikkelbroek Y Cuijpers P Berking M. Internet and computer-based cognitive behavioral therapy for anxiety and depression in youth: a meta-analysis of randomized controlled outcome trials. PLoS ONE. (2015) 10:e0119895. 10.1371/journal.pone.0119895 25786025 PMC4364968

[45] Wadwa RP Hanes S Clay M Weber I Forlenza G Buckingham B Impact of Early Initiation Of Continuous Glucose Monitoring on Glycemic Control in Pediatric Type 1 Diabetes Patients. Berlin: ATTD (2019).

[46] Toy BC Myung DJ He L Pan CK Chang RT Polkinhorne A. Smartphone-based dilated fundus photography and near visual acuity testing as inexpensive screening tools to detect referral warranted diabetic eye disease. Retina. (2016) 36:1000–8. 10.1097/IAE.0000000000000955 26807627

[47] Hanes SJ Wadwa RP Clay SM Weber I Forlenza G Buckingham BA CGM at diagnosis of type 1 diabetes: impact on glycemic and psychosocial outcomes. In: 12th International Conference on Advanced Technologies and Treatments for Diabetes. Berlin (2019).

[48] Swift PG Skinner TC De Beaufort CE Cameron FJ Aman J Aanstoot HJ. Target setting in intensive insulin management is associated with metabolic control: the Hvidoere childhood diabetes study group centre differences study 2005. Pediatr Diabetes. (2010) 11:271–8. 10.1111/j.1399-5448.2009.00596.x 19895567

[49] Battelino T Danne T Bergenstal RM Amiel SA Beck R Biester T. Clinical targets for continuous glucose monitoring data interpretation: recommendations from the international consensus on time in range. Diabetes Care. (2019) 42:1593–603. 10.2337/dci19-0028 31177185 PMC6973648

[50] Bergenstal RM Beck RW Close KL Grunberger G Sacks DB Kowalski A. Glucose Management Indicator (GMI): a new term for estimating A1C from continuous glucose monitoring. Diabetes Care. (2018) 41:2275–80. 10.2337/dc18-1581 30224348 PMC6196826

[51] DiMeglio LA Acerini CL Codner E Craig ME Hofer SE Pillay K ISPAD Clinical Practice Consensus Guidelines 2018: glycemic control targets and glucose monitoring for children, adolescents, and young adults with diabetes. Pediatr Diabetes. (2018) 19(Suppl. 27):105–14. 10.1111/pedi.12737 30058221

[52] National Institute for Health and Care Excellence (UK) Diabetes (Type 1 and Type 2) in Children and Young People: Diagnosis and Management. Diabetes (Type 1 and Type 2) in Children and Young People: Diagnosis and Management (London). (2015). 26334077

[53] Chiang JL Maahs DM Garvey KC Hood KK Laffel LM Weinzimer SA. Type 1 diabetes in children and adolescents: a position statement by the american diabetes association. Diabetes Care. (2018) 41:2026–44. 10.2337/dci18-0023 30093549 PMC6105320

[54] Danne T Nimri R Battelino T Bergenstal RM Close KL Devries JH. International consensus on use of continuous glucose monitoring. Diabetes Care. (2017) 40:1631–40. 10.2337/dc17-1600 29162583 PMC6467165

[55] Abraham MB Jones TW Naranjo D Karges B Oduwole A Tauschmann M. Assessment and management of hypoglycemia in children and adolescents with diabetes. Pediatr Diabetes. (2018) 27:178–92. 10.1111/pedi.12698 29869358

[56] Wong JC Neinstein AB Spindler M Adi S. A minority of patients with type 1 diabetes routinely downloads and retrospectively reviews device data. Diabetes Technol Ther. (2015) 17:555–62. 10.1089/dia.2014.0413 26133226 PMC4529086

[57] Nathan DM Kuenen J Borg R Zheng H Schoenfeld D Heine RJ. Translating the A1C assay into estimated average glucose values. Diabetes Care. (2008) 31:1473–8. 10.2337/dc08-0545 18540046 PMC2742903

[58] Juvenile Diabetes Research Foundation Continuous Glucose Monitoring Study G Beck RW Hirsch IB Laffel L Tamborlane WV Bode BW The effect of continuous glucose monitoring in well-controlled type 1 diabetes. Diabetes Care. (2009) 32:1378–83. 10.2337/dc09-0108 19429875 PMC2713649

[59] Esteva A Brett KB Novoa RA Ko J Swetter SM Blau HM. Dermatologist-level classification of skin cancer with deep neural networks. Nature. (2017) 542:115–8. 10.1038/nature21056 28117445 PMC8382232

[60] Biester T Nir J Remus K Farfel A Muller I Biester S. DREAM5: an open-label, randomized, cross-over study to evaluate the safety and efficacy of day and night closed-loop control by comparing the MD-Logic automated insulin delivery system to sensor augmented pump therapy in patients with type 1 diabetes at home. Diabetes Obes Metab. (2018) 21:822–8. 10.1111/dom.13585 30478937

[61] Nimri R Dassau E Segall T Muller I Bratina N Kordonouri O. Adjusting insulin doses in patients with type 1 diabetes who use insulin pump and continuous glucose monitoring: Variations among countries and physicians. Diabetes Obes Metab. (2018) 20:2458–66. 10.1111/dom.13408 29885025

[62] Chimen M Kennedy A Nirantharakumar K Pang TT Andrews R Narendran P. What are the health benefits of physical activity in type 1 diabetes mellitus? A literature review. Diabetologia. (2012) 55:542–51. 10.1007/s00125-011-2403-2 22189486

[63] Brouwer SI Stolk RP Liem ET Lemmink KA Corpeleijn E. The role of fitness in the association between fatness and cardiometabolic risk from childhood to adolescence. Pediatr Diabetes. (2013) 14:57–65. 10.1111/j.1399-5448.2012.00893.x 22830519

[64] Dunton GF Huh J Leventhal AM Riggs N Hedeker D Spruijt-Metz D. Momentary assessment of affect, physical feeling states, and physical activity in children. Health Psychol. (2014) 33:255–63. 10.1037/a0032640 23668846 PMC4113469

[65] McCarthy MM Funk M Grey M. Cardiovascular health in adults with type 1 diabetes. Prev Med. (2016) 91:138–43. 10.1016/j.ypmed.2016.08.019 27527572 PMC5050146

[66] Matson RIB Leary SD Cooper AR Thompson C Narendran P Andrews RC. Objective measurement of physical activity in adults with newly diagnosed type 1 diabetes and healthy individuals. Front Public Health. (2018) 6:360. 10.3389/fpubh.2018.00360 30581813 PMC6293090

[67] Sundberg F Forsander G Fasth A Ekelund U. Children younger than 7 years with type 1 diabetes are less physically active than healthy controls. Acta Paediatr. (2012) 101:1164–9. 10.1111/j.1651-2227.2012.02803.x 22849395

[68] Brazeau AS Leroux C Mircescu H Rabasa-Lhoret R. Physical activity level and body composition among adults with type 1 diabetes. Diabet Med. (2012) 29:e402–8. 10.1111/j.1464-5491.2012.03757.x 22817453

[69] Colberg SR Laan R Dassau E Kerr D. Physical activity and type 1 diabetes: time for a rewire? J Diabetes Sci Technol. (2015) 9:609–18. 10.1177/1932296814566231 25568144 PMC4604550

[70] Riddell MC Gallen IW Smart CE Taplin CE Adolfsson P Lumb AN. Exercise management in type 1 diabetes: a consensus statement. Lancet Diabetes Endocrinol. (2017) 5:377–90. 10.1016/S2213-8587(17)30014-1 28126459

[71] Yardley JE Brockman NK Bracken RM. Could age, sex and physical fitness affect blood glucose responses to exercise in type 1 diabetes? Front Endocrinol. (2018) 9:674. 10.3389/fendo.2018.00674 30524371 PMC6262398

[72] Chetty T Shetty V Fournier PA Adolfsson P Jones TW Davis EA. Exercise management for young people with type 1 diabetes: a structured approach to the exercise consultation. Front Endocrinol. (2019) 10:326. 10.3389/fendo.2019.00326 31258513 PMC6587067

[73] Prahalad P Ding V Addala A New C Conrad B Chmielewski A Early CGM initiation improves HbA1c in T1D youth over the first 15 months. In: American Diabetes Association 80th Scientific Sessions Submitted Abstract. Chicago, IL (2020).

[74] Lee MY Leverenz J Pageler N Scheinker D Maahs DM Prahalad P Pilot study on the feasibility of weekly CGM data review in pediatric patients with type 1 diabetes. American Diabetes Association 80th Scientific Sessions - Submitted Abstract. Chicago.

[75] Phillip M Battelino T Atlas E Kordonouri O Bratina N Miller S. Nocturnal glucose control with an artificial pancreas at a diabetes camp. N Engl J Med. (2013) 368:824–33. 10.1056/NEJMoa1206881 23445093

[76] Russell SJ El-Khatib FH Sinha M Magyar KL Mckeon K Goergen LG. Outpatient glycemic control with a bionic pancreas in type 1 diabetes. N Engl J Med. (2014) 371:313–25. 10.1056/NEJMoa1314474 24931572 PMC4183762

[77] Thabit H Tauschmann M Allen JM Leelarathna L Hartnell S Wilinska ME. Home use of an artificial beta cell in type 1 diabetes. N Engl J Med. (2015) 373:2129–40. 10.1056/NEJMoa1509351 26379095 PMC4697362

[78] Brown SA Kovatchev BP Raghinaru D Lum JW Buckingham BA Kudva YC. Six-month randomized, multicenter trial of closed-loop control in type 1 diabetes. N Engl J Med. (2019) 381:1707–17. 10.1056/NEJMoa1907863 31618560 PMC7076915

